# Impact of the Diagnosis-Intervention Packet Payment Reform on Provider Behavior in China: A Controlled Interrupted Time Series Study

**DOI:** 10.34172/ijhpm.8463

**Published:** 2024-12-19

**Authors:** Ruixin Wang, Jiaqi Yan, Xinyu Zhang, Mengcen Qian, Xiaohua Ying

**Affiliations:** ^1^School of Public Health, Fudan University, Shanghai, China.; ^2^Key Laboratory of Health Technology Assessment (Fudan University), Ministry of Health, Shanghai, China.

**Keywords:** Diagnosis-Intervention Packet, Payment Reform, Provider Behavior, Interrupted Time Series, China

## Abstract

**Background:**

China has developed a novel case-based payment method called the diagnosis-intervention packet (DIP) to regulate healthcare providers’ behavior. G city, a metropolis in southeast China, has shifted its payment policy from fixed rate per admission to DIP under regional global budget since 2018. This study examined the immediate and trend changes in provider behavior after this payment reform.

**Methods:**

Discharge data in G city between 2016 and 2019 was used, covering more than 10 million inpatient cases in 320 hospitals. A counterfactual scenario was developed to assign insured and uninsured inpatients across the study period to specific DIP groups under consistent rules. Controlled interrupted time series (ITS) analyses were performed, with uninsured inpatients as control. Outcomes included inpatient volume, average DIP weight (similar to case-mix index [CMI] in diagnosis-related groups [DRGs]), and two innovative indicators (average diagnostic weight and average treatment weight) to decompose the changes in DIP weight. Subgroup analyses were conducted for different hospital levels and 21 major disease categories.

**Results:**

After the DIP reform, monthly trend of inpatient volume decreased (-1085.34, *P*=.052), while monthly growth of average DIP weight increased (2.17, *P*=.02). No significant changes in average diagnostic weight were observed. Monthly trend of average treatment weight increased (2.38, *P*=.001) after the reform. Secondary and tertiary hospitals experienced insignificantly decreased inpatient volume and elevated average DIP weight, accompanied by negligible change in average diagnostic weight and significant increase in average treatment weight. Primary hospitals experienced reduced inpatient volume and stable average DIP weight, along with increase in average diagnostic weight and decrease in average treatment weight.

**Conclusion:**

By differentiated payments for severity, DIP induced hospitals to shift their focus from volume to weight of inpatients. Instead of diagnostic upcoding, hospitals responded to the DIP reform primarily by increasing treatment intensity. Primary hospitals may face financial risks under regional competition.

## Background

**Key Messages**

**Implications for policy makers**

- By adopting differentiated payment, the diagnosis-intervention packet (DIP) reform induced hospitals to shift their focus from volume to complexity of inpatients.
- The average DIP weight can be decomposed to identify unusual changes in diagnosis and treatment behavior among providers.
- Instead of diagnostic upcoding, hospitals responded to the DIP reform primarily by increasing treatment intensity, especially in specific disease categories with multiple treatment choices.
- Although DIP mitigates the drawbacks of conventional cost containment strategies to some extent, policy-makers should be wary of supplier-induced demand and potential inefficiency.
- DIP is supposed to be combined with regional global budget to curb unreasonable cost increase, while primary hospitals that have relatively weak capacity to adapt to the reform may face financial risks under the regional competition.

**Implications for the public**

China has been exploring effective payment methods to induce hospitals to curb unnecessary health expenditure. A novel approach called diagnosis-intervention packet (DIP) was developed, which reimbursed hospitals proportional to the complexity of principal diagnosis and treatments delivered, under a predetermined municipal total budget. We found that the reform induced hospitals to shift their attention from the volume of hospitalization to the severity of inpatients. Hospitals responded to the DIP reform primarily by increasing treatment intensity, especially in specific disease categories with multiple treatment choices. Primary hospitals have relatively weak capacity to adapt to the reform and may face financial risks under the regional competition. Our findings suggested that the DIP could potentially mitigate the drawbacks of undertreatment among conventional cost control strategies. Policy-makers could develop complementary measures to reduce potential risks and improve this promising strategy, to approach a more efficient healthcare system which we can all benefit from.

Rising healthcare costs have been a major concern for the healthcare system in China. Health expenditure has seen an annual increase of 14.61% over the last two decades, significantly outpacing the growth of gross domestic product at 12.38% during the same period.^1^ In addition to an aging population and advancements in medical technology, the reimbursement system for healthcare providers plays a crucial role in driving up healthcare costs.^2^ To contain costs and improve efficiency in healthcare delivery, China has developed a novel case-based payment system called the diagnosis-intervention packet (DIP), combined with a regional global budget scheme. Since 2020, this payment system has been implemented in more than 70 cities across China.

 G city is one of the largest and most economically developed cities in southeast China, with a population of over 18.7 million in 2022.^3^ The social insurance system in G city consists of two main components: the Urban Employee Basic Medical Insurance scheme and the urban and rural resident basic medical insurance scheme, which was established in 2015 by integrating the original Urban Resident Basic Medical Insurance scheme with the new cooperative medical scheme. In 2015, G city implemented a stringent cost-control policy that involved assigning a fixed rate per admission and setting an annual compensation ceiling for insured patients at hospitals participating in the social insurance program. These rates and ceilings were specific to each hospital. While this approach effectively managed to control medical expenditure, it may incentivize hospitals to adopt patients with less severe conditions and provide minimal services.

 In January 2018, G city was one of the first pilot cities to implement the DIP reform, replacing the previous payment system for insured inpatients. Similar to diagnosis-related group (DRG) systems, the DIP is based on a patient classification approach. However, the grouping approach of DIP is more refined and data-driven. Discharge records from the past three years were used to cluster patients according to the actual combination of principal diagnosis (four-digit ICD-10 [the International Classification of Diseases, Tenth Revision] code) and treatments (based on ICD9-CM-3). This resulted in a more comprehensive set of DIP groups, with over 12 000 identified in G city, compared to less than 1000 groups under the DRG systems elsewhere in China.

 As a prospective payment system, the DIP revised the incentive structure for hospitals’ medical service delivery. Each DIP group had a specific relative weight (RW) set at the city level, based on the average health expenditure of inpatients in the last three years. The RW for a given DIP group was determined relative to the average cost for laparoscopic appendectomy, which had a default value of 1000.^4^ The crude weight would be adjusted by factors such as patient age and hospital level during the final accounts period.

 The total RWs for insured inpatients in a given hospital, representing the level of resource consumption based on the diagnosis-treatment combination, can be converted into the annual insurance reimbursement for the hospital by multiplying it by the base rate (BR). The BR represents the payment price for each RW under the DIP system, which is identical for all hospitals in G city. It is a floating rate, set at the end of the year by the G city Medical Insurance Bureau, to align with the overall global budget. The budget was set in advance at the city level and divided by the annual sum of DIP weights of all the insured inpatient cases in G city.^5^

 Both the RW of a specific DIP group and the BR were set at the municipal level, which could not be manipulated by individual hospitals. Theoretically, hospitals could simply accept the market price. In practice, however, hospitals may change their delivery behavior in response to the DIP reform. To obtain more reimbursement under the regional global budget, hospitals were supposed to pursue higher annual total DIP weights, as it led to a larger share when competing with other hospitals. Two strategies can be employed for hospitals to enhance their total weights: adopting more inpatients and/or enhancing the average DIP weight of inpatients. The second strategy could take various forms, such as admitting patients with more severe conditions, providing more advanced treatment, or manipulating information in medical records to classify patients into higher-reimbursement groups.^6,7^

 Regarding the first strategy, previous literature showed mixed results regarding the impacts of DIP or the analogous DRG system on inpatient volume. Lai found no measurable changes in admission and readmission volume after the DIP reform among insured local inpatients relative to non-local inpatients.^8^ Similarly, Al-Khalil et al found no evidence of any effect of Swiss-DRG on the annual trend of primary care consultations numbers in Switzerland.^9^ However, a city in central China experienced a statistically significant reduction in inpatient volume of 14.3% after DIP implementation compared to other cities in the same province.^10^ In the United States, hospital utilization declined after the DRG-prospective payment system reform, and the focus of healthcare shifted from inpatients to less costly outpatient settings.^11^

 For the second strategy, prior studies on DIP showed an overall increase in average weight and mixed findings in the service delivery behind. A difference-in-differences analysis found that average point volume per case increased by more than 3% after the DIP reform, coupled with increasing likelihood of performing at least one procedure and decrease in drug expenditures and total health expenditures per case.^8^ One study showed that proportion of anti-infective drugs, anti-tumor drugs, and biological products decreased after the DIP reform, while the use of nutritional medications increased.^12^ Although some study found notable decrease of 9.1% in actual available bed days after DIP implementation,^10,13^ some found significant increase in length of stay,^14,15^ and others found it varied across patients^16^ and hospitals.^17^ With regard to patient composition, one recent study found that the differences in patient severity grew after the DIP reform,^18^ whereas another previous study identified no significant change in the age-adjusted Charlson index after the DIP payment.^8^ In terms of diagnostic upcoding, which has been widely identified as an unintended consequence of the classic DRG system,^19-23^ one study provided suggestive evidence of up-coding in response to the DIP reform, but the finding has yet to be confirmed.

 Overall, the existing literature has provided inconclusive evidence on the specific behavioral changes of hospitals in response to the DIP reform. To address this gap, this study proposed an innovative approach to decompose the overall case-mix into two dimensions of diagnostic and treatment, and employed a controlled interrupted time series (ITS) design to assess the impact of the DIP reform on provider behavior, with a specific focus on patient admission, diagnostic coding, and treatment delivery. Understanding providers’ response to DIP is crucial for policy-makers to design effective strategies to mitigate potential strategic behaviors and ensure the successful implementation of the payment reform. The findings from this study will contribute to the growing body of literature on the impact of alternative payment models on provider behavior and healthcare delivery.

## Methods

###  Data

 We obtained de-identified claim data in G city from 2016 to 2019, covering over 10 million inpatient cases. Each record represents a hospitalization and includes variables such as date of admission and discharge, patient characteristics (age, gender, and insurance status), hospital characteristics (level, ownership, and location), primary diagnosis and up to 14 secondary diagnoses (ICD-10 codes), primary procedure and up to 7 secondary procedures (ICD-9-CM-3 codes), and health expenditures (total, diagnosis, treatment, drugs, consumables, blood, rehabilitation, out-of-pocket, etc). For insurance status, insured inpatients were those with local health insurance in G city, while uninsured inpatients were those with insurance from elsewhere or without any insurance. To facilitate time series analyses, we aggregated the discharge-level data into monthly statistics for insured and uninsured inpatients at the municipal level.

 Additionally, official data on DIP groups and weights was acquired from the Health Insurance Bureau, covering over 12 000 DIP groups. In practice, the grouping rules and weights updated annually, which means a given case might be assigned to different DIP groups and weights between 2018 and 2019. To investigate the effects of DIP reform, we constructeda counterfactual scenario by classifying all the inpatient cases from 2016 to 2019 according to the grouping rules and weights in 2018. Therefore, pre-DIP cases (2016-2017), patients uncovered by DIP (uninsured inpatients), and patients involved in different versions of DIP (insured inpatients, 2018-2019) could have consistent DIP groups and weights for a given combination of diagnosis and procedures.

###  Measures

 Four outcome indicators were used to capture healthcare behavioral changes after the DIP payment reform.

1. Inpatient volume: As an indicator of health services utilization, inpatient volume could reflect the supply of medical services in hospitals, based on the assumption that the medical demand of all patients in a given city did not change within a short timeframe.
2. Average DIP weight: The average DIP weight was used to measure the overall complexity and resource intensity of inpatients. It is a regional aggregate indicator, calculated as the weighted average of the RW and cases of each DIP group. This indicator is analogous to the case-mix index (CMI) in DRG, which is generated by the health insurance sector to evaluate service delivery and determine reimbursement to hospitals. A higher CMI or average DIP weight suggests more complex cases or more adoption of advanced treatment.^24,25^ Assuming that regional disease patterns and health technology advancements should steadily follow a natural grow trajectory within a short timeframe before and after the reform, changes in average DIP weight can signal an overall healthcare behavioral change in response to the DIP reform.
3. Average diagnostic weight: The average diagnostic weight was used to assess diagnostic behavior within overall healthcare service delivery. We started by keeping the composition of various treatments fixed for each diagnosis and then observed changes in the composition of different diagnoses over time. First, we kept the composition of various treatments before the DIP reform (2016-2017) and calculated the average DIP weights for each diagnosis, referred to as “diagnostic weight.” This measurement reflected each diagnosis’s level of resource consumption before the DIP reform and was considered a fixed property of each diagnosis. Next, we combined the time-varying monthly cases of each diagnosis with this fixed “diagnostic weight” to calculate the “average diagnostic weight” in G city for each month. Assuming that the natural composition of disease diagnoses remained stable in G city within a short timeframe, abnormal growth in average diagnostic weight may indicate systematic changes in diagnostic coding behavior among healthcare providers.
4. Average treatment weight: The average treatment weight was used to assess treatment behavior within overall healthcare service delivery. We started by calculating the average DIP weight within each diagnosis for each month, referred to as “treatment weight.” Next, we calculated the proportion of each diagnosis among total inpatients during 2016-2017 (pre-DIP). Finally, we combined the pre-DIP composition of diagnoses with the monthly-varying “treatment weight” to compute the “average treatment weight” in G city for each month. The “treatment weight” reflected the monthly level of resource consumption across treatments within each diagnosis. The “average treatment weight” projected these treatment variations onto a fixed distribution of diagnoses, indicating overall utilization of advanced treatments in G city in each month. Assuming steady health technology advances within a short timeframe, aberrant growth in average treatment weight may indicate systematic changes in supply of advanced treatments.

To sum up, average DIP weight reflects the overall case-mix of inpatients, and its change can be caused by an increase or decrease in either average diagnostic weight or average treatment weight. The changes in average diagnostic weight and average treatment weight are independent of each other, as the former is determined by the case proportion of patients with different diagnoses, while the latter is determined by the case proportion of patients adopting different treatments within each diagnosis (with the composition of diagnoses remains at the pre-DIP level). A numerical example for weight calculation is provided in Supplementary file 1.

###  Statistical Analysis

 Controlled ITS analyses^26^ were performed to assess the immediate and trend effects of the DIP reform. The treatment group included insured inpatients in G city, as they were target population of the DIP payment policy. The control group comprised uninsured patients hospitalized during the same period. Monthly data at the municipal level was used, and January 2018 was set as the starting point of the DIP intervention. The model was specified as:

 Y_t_ = *β*_0_ + *β*_1_*T*_t_ + *β*_2_*DIP*_t _+ *β*_3_*DIP*_t_*T*_t _+ *β*_4_*Insured *+ *β*_5_*InsuredT*_t _+ *β*_6_*InsuredDIP*_t _+ *β*_7_*InsuredDIP*_t_*T*_t _+ *α X*_t_ + *ɛ*_t_

 where *Y*_t_ represents the aggregated outcomes in G city in month t, including inpatient volume, average DIP weight, average diagnostic weight, and average treatment weight. *T*_t_is a continuous month count from January 2016 to December 2019. *DIP*_t_ is a dummy variable, which equals 0 before the DIP reform and equals 1 after the reform. *Insured* is a dummy variable, which equals 0 for uninsured inpatients and equals 1 for insured inpatients. *X*_t_ denotes a series of aggregated covariates, including average age, proportion of male, average Charlson Comorbidity Index, and seasonality. *ɛ*_t_ is the error term. The key effect estimates were *β*_6_ and *β*_7_, which estimated the immediate and trend change of the DIP reform, respectively. Detailed explanation of the model can be found in Supplementary file 1.

 We fitted a Prais-Winsten estimation with the Durbin-Watson statistic to adjust for autocorrelation and used robust standard errors.^26,27^ We first conducted controlled ITS analysis for inpatients in all hospitals. Subsequently, subgroup analyses with the segmented regression model were conducted for hospitals of different levels and 21 major disease categories (based on ICD-10 coding system). Then we calculated the Pearson correlation coefficient between the estimated trend effects of average treatment weight and the number of DIP groups under each disease category, to investigate whether the disease categories with multiple treatment choices experienced a greater increase in average treatment weight after the DIP reform. The threshold of statistical significance was set at 0.05. Stata 17 was used for all analyses.

## Results

###  Sample Characteristics

 Our analysis included 10 378 151 discharge cases from 320 hospitals in G city, China, from 2016 to 2019. Table 1 presents the sample characteristics before and after the DIP payment reform for insured and uninsured patients, respectively. Insured patients accounted for two-thirds of the discharges. The average age of insured inpatients was around 54 years old, while 40 years old among uninsured inpatients. For both groups, about 45% of the sample were male inpatients. Most cases were from tertiary and public hospitals, with about 80% and 94%, respectively. After the DIP reform, there was an increase in monthly inpatient volume and average diagnostic weight among insured patients and decrease among the uninsured. Both groups experienced an increase in average DIP weight and average treatment weight.

**Table 1:** Descriptive Statistics Before and After the Diagnosis-Intervention Packet Reform Among Insured and Uninsured Patients

	**Before DIP Reform (2016-2017)**		**After DIP Reform (2018-2019)**	
	**Insured**	**Uninsured**	**Insured**	**Uninsured**
**Outcome Variables**				
Monthly inpatient volume, mean (SD)	101 903 (16 601)	47 826 (6458)	120 512 (15 844)	44 295 (8100)
Average DIP weight, mean (SD)	1011.63 (930.58)	924.49 (881.43)	1133.63 (1106.92)	942.34 (920.35)
Average diagnostic weight, mean (SD)	1008.11 (616.39)	912.08 (628.46)	1064.96 (637.02)	900.36 (617.05)
Average treatment weight, mean (SD)	1005.30 (0.34)	913.44 (0.49)	1049.95 (0.38)	929.14 (0.52)
**Patient Characteristics**				
Age, mean (SD)	53.82 (21.55)	40.44 (20.91)	53.90 (21.86)	38.52 (21.63)
Gender, No. (%)				
Male	1 456 568 (45.78)	746 336 (44.19)	1 669 452 (46.23)	666 939 (43.62)
Female	1 725 416 (54.22)	942 610 (55.81)	1 942 029 (53.77)	862 123 (56.38)
Charlson Comorbidity Index, No. (%)				
0	1 647 310 (62.11)	960 989 (77.58)	1 947 671 (58.46)	1 004 105 (79.25)
1	514 689 (19.41)	133 067 (10.74)	631 464 (18.95)	118 631 (9.36)
2	311 413 (11.74)	84 568 (6.83)	460 698 (13.83)	83 795 (6.61)
≥3	178 895 (6.74)	60 081 (4.85)	291 878 (8.76)	60 505 (4.78)
Hospital level, No. (%)				
Tertiary (N = 94)	2 596 283 (81.06)	1 319 974 (77.54)	3074 026 (80.60)	1 257 213 (77.19)
Secondary (N = 95)	468 097 (14.62)	323 821 (19.02)	535 434 (14.04)	306 628 (18.83)
Primary (N = 131)	138 410 (4.32)	58 567 (3.44)	204 524 (5.36)	64 932 (3.99)
Hospital ownership, No. (%)				
Public (N = 246)	3 067 343 (95.78)	1 600 063 (93.99)	3 652 994 (95.35)	1 515 581 (92.39)
Private (N = 62)	135 009 (4.22)	102 280 (6.01)	178 334 (4.65)	124 816 (7.61)
Sample size	3 202 790	1 702 368	3 832 471	1 640 522

Abbreviations: DIP, diagnosis-intervention packet; SD, standard deviation. N denoted the number of hospitals.

###  Inpatient Volume

**Figure 1:**
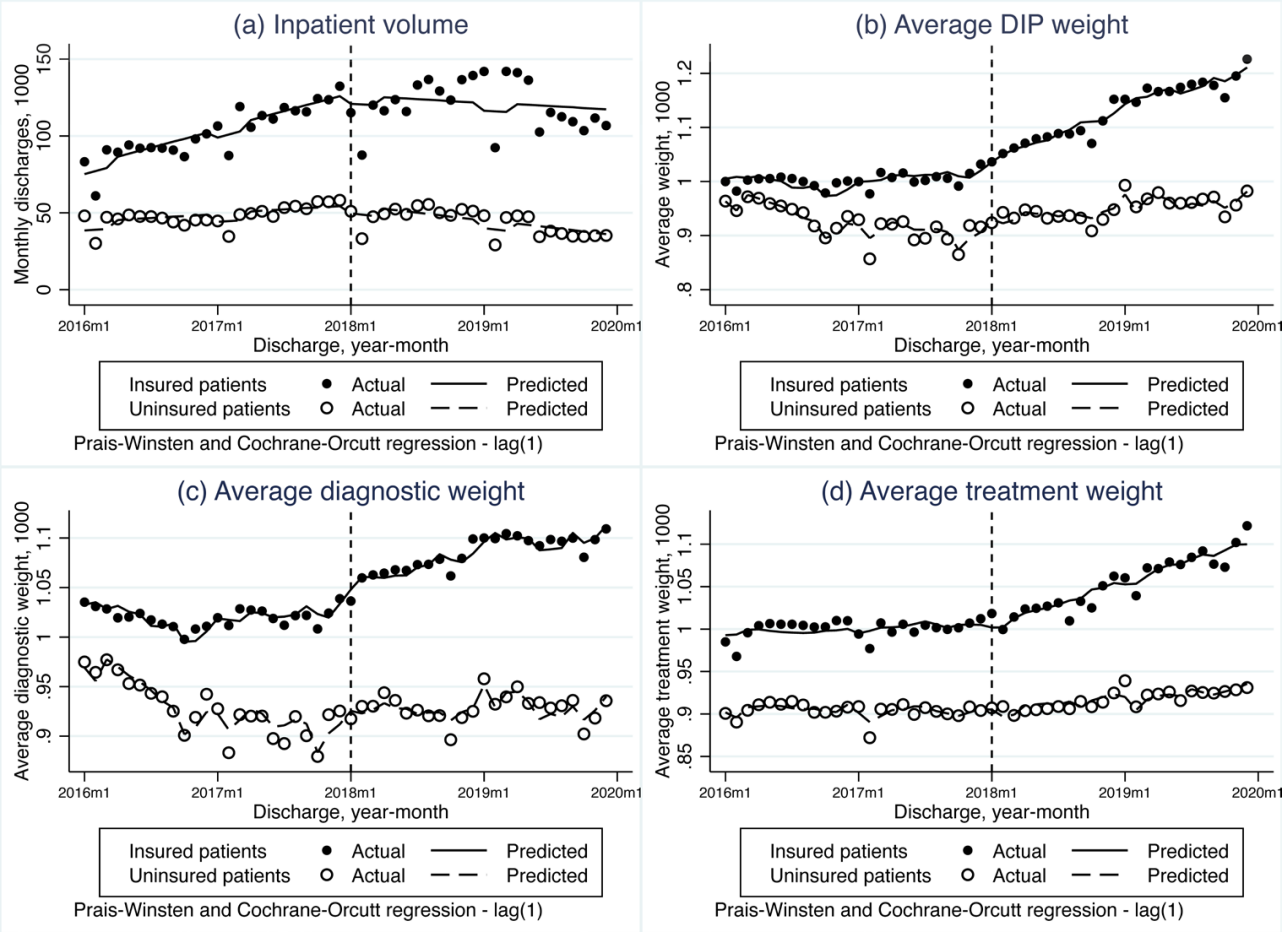
Monthly Changes in Inpatient Volume, Average DIP weight, Average Diagnostic Weight, and Average Treatment Weight in Insured Inpatients Compared to Uninsured Inpatients Among All the Hospitals in G City, 2016-2019. Abbreviation: DIP, diagnosis-intervention packet.

Table 2 shows the immediate and trend effects of the DIP payment reform based on controlled ITS analysis, for the whole sample and subgroups by hospital level, respectively. Before the DIP reform, the growth rate of inpatient volume among insured patients was significantly higher than that among uninsured patients, with a difference of 1516.62 cases in monthly slope (*P*< .001, Table S1, Supplementary file 1). After the DIP payment, there were no immediate changes in inpatient volume of insured patients in comparison with uninsured patients, for both overall and subgroup analyses. The monthly trend of overall inpatient volume in G city decreased (-1085.34 cases per month, *P*= .052) and specifically among primary hospitals (-222.49 cases per month, *P*< .001). Figure 1a presents the overall effects of the DIP reform on inpatient volume. Figure S1a, Figure S2a and Figure S3a present the effects for hospitals with different levels.

**Table 2:** Controlled Interrupted Time Series Estimates for Diagnosis-Intervention Packet Payment Reform in G City

	**Difference in Step Change Between Groups (β6)**			**Difference in Trend Change Between Groups (β7)**		
	**Estimate**	**(95% CI)**	***P*** ** Value**	**Estimate**	**(95% CI)**	***P*** ** Value**
Overall						
Inpatient volume	-1800.75	(-16 740.03, 13 138.54)	.81	-1085.34	(-2182.47, 11.79)	.052
Average DIP weight	-2.68	(-26.36, 21.00)	.82	2.17	(0.31, 4.03)	.023
Average diagnostic weight	5.45	(-13.67, 24.57)	.57	-0.26	(-1.70, 1.18)	.72
Average treatment weight	0.73	(-14.70, 16.16)	.93	2.38	(0.99, 3.78)	.001
Tertiary hospitals						
Inpatient volume	-2992.90	(-16 023.67, 10 037.87)	.65	-842.20	(-1815.40, 130.99)	.09
Average DIP weight	-13.42	(-34.34, 7.50)	.21	2.14	(0.68, 3.61)	.005
Average diagnostic weight	-0.26	(-17.34, 16.81)	.98	-0.15	(-1.29, 1.00)	.80
Average treatment weight	-3.14	(-17.38, 11.10)	.66	2.35	(1.28, 3.43)	0.001
Secondary hospitals						
Inpatient volume	1119.83	(-1119.05, 3358.70)	.32	-24.62	(-186.22, 136.99)	.76
Average DIP weight	12.06	(-12.22, 36.34)	.33	3.19	(1.01, 5.38)	.005
Average diagnostic weight	18.55	(-2.66, 39.76)	.09	1.52	(-0.03, 3.07)	.054
Average treatment weight	17.91	(-2.56, 38.39)	.09	2.82	(1.27, 4.36)	<.001
Primary hospitals						
Inpatient volume	35.08	(-796.34, 866.50)	.93	-222.49	(-283.78, -161.21)	<.001
Average DIP weight	4.18	(-52.47, 60.83)	.88	-0.40	(-4.83, 4.03)	.86
Average diagnostic weight	-40.15	(-68.01, -12.28)	.005	3.60	(0.92, 6.29)	.009
Average treatment weight	1.99	(-30.44, 34.41)	.90	-4.40	(-7.26, -1.54)	.003

Abbreviations: DIP, diagnosis-intervention packet; CI, confidence interval.

###  Average Diagnosis-Intervention Packet Weight

 Before the DIP reform, the average DIP weight slightly increased in insured patients and decreased in uninsured patients, and the difference in monthly trend between groups was significant (2.02 points per month, *P*= .007). After the DIP payment, there were no immediate changes in average DIP weight of insured inpatients compared to uninsured inpatients, for both overall and subgroup analyses (Table 2). The monthly trend of average weight among insured patients significantly increased in all hospitals (2.17 points per month, *P*= .02) compared to uninsured patients. Tertiary and secondary hospitals had similar trend change, with increasing slopes of 2.14 (*P*= .005) and 3.19 (*P*= .005), respectively. No significant change in average DIP weight among primary hospitals was observed (*P*= .86). Figure 1b, Figure S1b, Figure S2b and Figure S3b present the effects of the DIP reform on average DIP weight for the whole sample and hospitals with different levels, respectively.

###  Average Diagnostic Weight

 The average diagnostic weight fluctuated among insured patients and decreased among uninsured patients before the DIP reform (Table S1, Figure 1c), with an insignificant difference between groups of 1.02 points per month (*P*= .07). After the DIP reform, neither immediate change (*P*= .57) nor trend change (*P*= .72) in average diagnostic weight were observed when comparing insured patients with uninsured patients (Table 2). These findings were consistent in tertiary and secondary hospitals. The ITS estimates suggest that primary hospitals experienced decreased immediate change (-40.15, *P*= .005) and increased slope change (3.60, *P*= .009) in average diagnostic weight, while they may have subtle effects cumulatively. Figure S1c, Figure S2c and Figure S3c present the effects of the DIP reform on average diagnostic weight among hospitals with different levels.

###  Average Treatment Weight

 Before the DIP payment reform, the average treatment weight remained stable for both insured and uninsured inpatients, with differences in monthly trend between groups being insignificant (0.74 points per month, *P*= .16). After the adoption of DIP, there were no immediate changes in average treatment weight of insured inpatients compared to uninsured inpatients, for both overall and subgroup analyses (Table 2, Figure 1d, Figure S1d, Figure S2d, Figure S3d). Significant increase in monthly trend of average treatment weight was observed in all hospitals (2.38 points per month, *P*= .001), tertiary hospitals (2.35 points per month, *P*< .001) and secondary hospitals (2.82 points per month, *P*< .001). Conversely, the monthly slope of average treatment weight among insured patients in primary hospitals significantly decreased in comparison with those uninsured (-4.40 points per month, *P*= .003).

**Figure 2:**
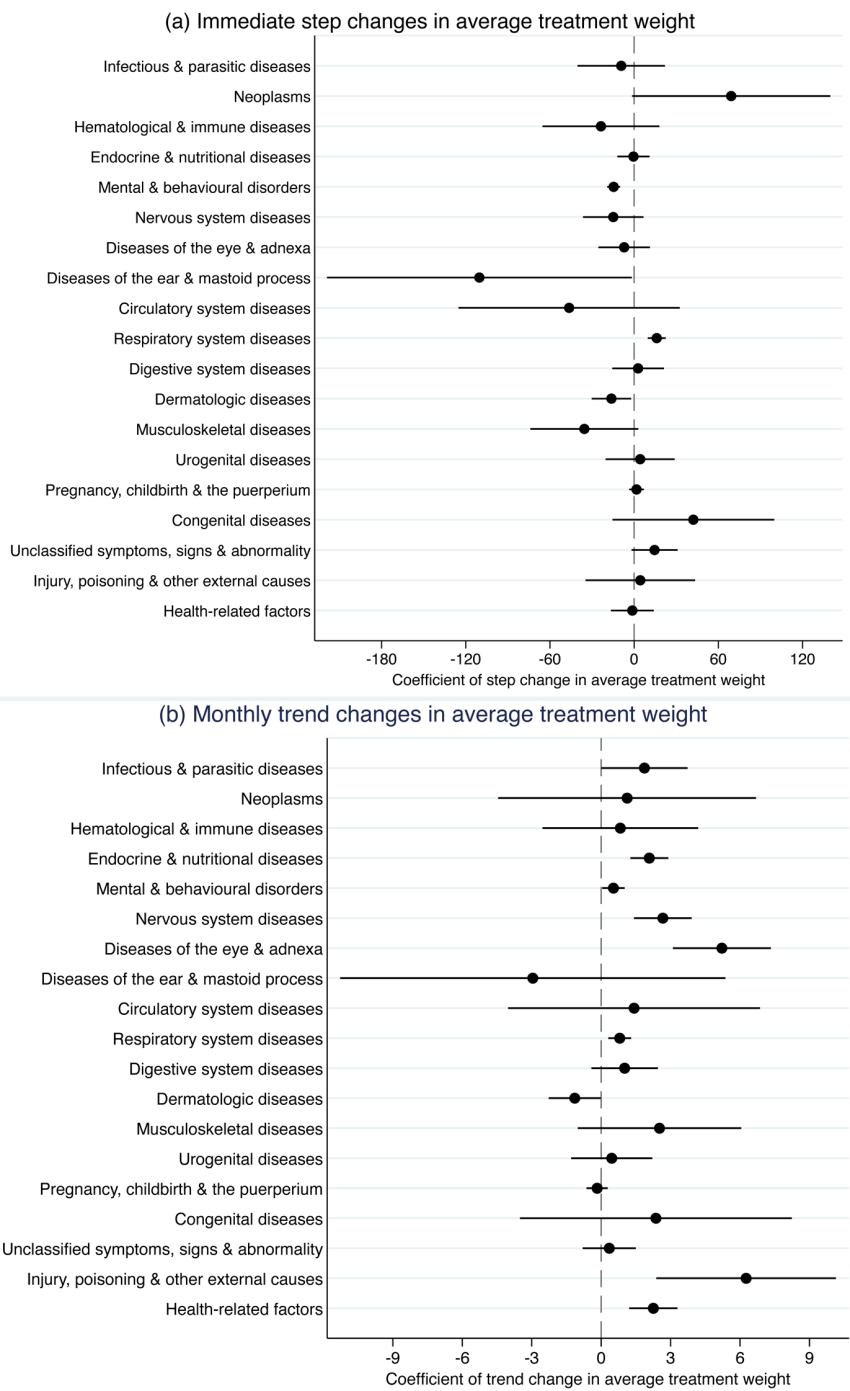
Immediate and Trend Changes in Average Treatment Weight Across Major Disease Categories After the Diagnosis-Intervention Packet Payment Reform in G City.

Figure 2 and Table S2 present the immediate and trend effects of the DIP reform on average treatment weight among 21 major disease categories. In terms of immediate effects, the DIP reform brought about elevated average treatment weights in respiratory system diseases (J00-J99) and decreased average treatment weight in mental and behavioral disorders (F00-F99), diseases of the ear and mastoid process (H60-H95), and diseases of the skin and subcutaneous tissue (L00-L99). In terms of trend effects, there was increased monthly growth in average treatment weight in certain infectious and parasitic diseases (A00-B99), endocrine, nutritional and metabolic diseases (E00-E90), mental and behavioral disorders (F00-F99), nervous system diseases (G00-G99), diseases of the eye and adnexa (H00-H59), respiratory system diseases (J00-J99), injury, poisoning and certain other consequences of external causes (S00-T98), and factors influencing health status and contact with health services (Z00-Z99). Among the 21 major disease categories according to the ICD-10 coding system, there were two disease categories (certain conditions originating in the perinatal period, and external causes of morbidity and mortality) having no inpatient cases in certain months during the study period, making it inappropriate to estimate the effects using controlled ITS analyses. The monthly changes in average treatment weight among these two disease categories were plotted in line charts (Figure S4). The descriptive graph showed relative increase in average treatment weight in certain conditions originating in the perinatal period (P00-P96) among insured inpatients compared to uninsured inpatients. Moreover, we found that among the disease categories whose average treatment weight experienced significant trend change after the DIP reform, there was a positive correlation between the number of DIP groups under each disease category (*r*= 0.61, *P*= .08). Although this correlation did not reach the established significance level of 0.05 in this study, it is noteworthy that it was significant at the 0.1 level, suggesting a potential relationship that may be considered acceptable within a broader context (Figure S5).

## Discussion

 This study investigated how healthcare providers change their diagnostic and treatment behavior when the health insurance payment policy shifted from fixed rate per admission to DIP payment under regional global budget. By adopting ITS analyses with uninsured inpatients as control, we found evidence of decreased inpatient volume, increased average DIP weight, stable average diagnostic weight and increased average treatment weight after the DIP reform. Furthermore, the impacts of the DIP payment reform vary across different hospitals and disease categories.

 Prior to the DIP reform, the payment approach in G city was fixed rate per admission, and patients with different conditions or severity were not reimbursed differentially. This strict cost-control system hampered the development and use of advanced medical technology.^28^ Since the reimbursement amount is determined mainly by the number of patients, hospitals tended to admit milder patients. This is consistent with our findings on the monthly trend before the DIP reform, where the inpatient volume continuously increased, and the average weight remained stable. Thus, the DIP policy was introduced to encourage adequate supply of essential medical services. We found reduction in the trend of patient volume (*P*= .052) and increase in growth of average DIP weight (*P*= .02) when shifting from a fixed rate to the DIP payment, suggesting that the hospitals shifted their focus from the number of inpatients to the weight of inpatients to get more reimbursement in the DIP payment system. This is consistent with the results from previous DIP studies. It was found that the average point volume per case (another name for “average DIP weight”) increased by more than 3% after the DIP reform.^8^ Similarly, evidence from another pilot city in China showed that the CMI in tertiary (β=0.022, *P*< .001) and secondary hospitals (β=0.008, *P*< .001) increased,^29^ and a difference-in-differences analysis found that RWs for arteriosclerotic heart disease patients in DIP hospitals increased 5.5% (*P*= .015) more than in non-DIP hospitals after payment reform.^18^

 As an indicator of proportion of various diagnoses, the average diagnostic weight exhibited insignificant changes after the DIP reform. This is consistent with our hypothesis that composition of disease diagnoses remains stable in G city in a short term before and after the DIP, suggesting limited changes in diagnostic coding behavior among healthcare providers. A prior study found suggestive evidence of up-coding that there was a positive correlation between the coefficient of variation of DIP weights within each major disease category and the estimated reform impact on the average weight.^8^ It might seem contradictory to our findings. However, the “up-coding” here referred to behavioral change under specific diseases, rather than the proportions of various diseases. Hence, the nature of this measure is much closer to the treatment weight in our study, which we have found the positive association similarly.

 After the DIP reform, the monthly growth of average DIP weight and average treatment weight increased, while average diagnostic weight barely changed. It indicates that the increase in average DIP weight mainly stemmed from more delivery of advanced treatments. Compared to the previous “fixed rate per admission” policy, the DIP system has differentiated reimbursement according to the complexity of inpatients. Providers were encouraged to provide more advanced treatments, as it could bring higher annual DIP weight and, accordingly, higher reimbursement. Prior study also found some evidence on elevated treatment intensity, by using various measurements. One study found that the share of inpatients who receive at least one procedure in Guangzhou has increased by more than 3 percentage points after the DIP implementation.^8^ An ITS analysis based on another DIP pilot city founded that the proportion of patients receiving complicated procedures in tertiary (β = 0.197, *P* < .001) and secondary hospitals (β = 0.132, *P*= .020) increased.^29^ These two measurements of treatment intensity are more intuitive than average treatment weight but have their limitations. The “share of inpatients who receive at least one procedure” is unable to capture the change in treatment intensity in cases where cheaper procedure is replaced by more advanced procedure, or cases where single procedure is replaced by multiple procedure. The “proportion of patients receiving complicated procedures” bisect all the cases, making it impossible to identify the changes in each subset (ie, cases where a more advanced treatment is used, but it is still in the “non-complicated procedures” section; or cases where two complicated procedures are used instead of one complicated procedure). The average treatment weight is advantageous for capturing all these kinds of changes in treatment intensity in a way undisturbed by the confounding of changing diagnostic composition.

 We also found a potentially positive correlation between the number of DIP groups under each disease category and the estimated trend effects of average treatment weight, suggesting that the increasing treatment intensity is more likely to happen in disease categories with multiple treatment choices. The enhanced treatment intensity may further bring about improvement in quality of care, especially among severe inpatients who might be under-reimbursed in the previous strict fixed-rate policy. It was found a 3.6% reduction (*P*= .046) in postoperative complication rate in response to DIP adoption among severe patients.^30^

 In the subgroup analyses, reduction in inpatient volume across various levels of hospitals was observed after the DIP reform, although only significant in primary hospitals. Similarly, another pilot city in a central province in China experienced a reduction in inpatient volume of 14.3% after DIP implementation, and primary hospitals experienced the greatest impact, with a 19.0% decline in inpatient volume.^10^ In China, the absence of gatekeeper systems, combined with the inadequate quality of primary healthcare facilities, frequently results in patients with minor diseases bypassing primary healthcare facilities and directly seeking care in superior hospitals.^31-36^ After the DIP reform, tertiary and secondary hospitals continue to attract patients from primary hospitals owing to their superior medical capabilities, resulting in a significant decrease in patient volume at primary hospitals.^17^ One prior study from Tai’an in eastern China found that after the DIP reform, tertiary and secondary hospitals have taken on more severe treatment tasks, primary hospitals adopted fewer patients with minor illnesses and experienced increasing CMI.^29^ According to the head of the Tai’an Healthcare Security Administration: “The service capabilities of primary healthcare facilities in Tai’an are still quite restricted. Rather than assuming the risks associated with treating severe cases, they are more inclined toward upcoding due to the comparatively lower penalties involved.”^29^ This corresponded to our finding of increased growth in average DIP weight in tertiary and secondary hospitals, as well as elevated average diagnostic weight and decreased average treatment weight in primary hospitals. The interview with different levels of hospitals in G city showed that it was challenging for primary hospitals to adapt to the DIP reform due to lack of medical insurance offices, experienced administrative staff and coding specialists. Considering the significant reduction of inpatient volume and stable average weight, primary hospitals might receive reduced reimbursement in “DIP weight” competition under the global budget settings. Potential supportive measures include setting differentiated adjustment coefficient for hospitals of different levels, introducing primary DIP groups to differentiate diseases supposed to be treated in primary hospitals, “two lines of revenue and expenditure” policy (the government provides complete funding for the expenses of primary healthcare facilities, and consequently, all revenue generated by these institutions is remitted to the government) with caution on productivity reduction, and dynamic adjustments to primary DIP groups.^29^

 Additional key factors that could affect the DIP weight were addressed to confirm that the increase in average weight was caused by the actual behavioral changes in the hospitals. Previous research concluded that the change in average weight might be caused by medical practice changes, aging of the inpatient population, changes in coding practices of physicians and hospitals, and changes in the way that the Health Care Financing Administration collects data on the weights.^37^ First, inpatient aging can be excluded, as the descriptive analysis showed that the changes in average age from 2016 to 2019 was unremarkable. Second, a counterfactual scenario was developed to classify patients into DIP groups consistently according to the grouping rules in 2018. Therefore, changes in the data collection process can be excluded. Third, the shift in average weight could not be attributed to coding behavioral change, as we used uninsured inpatients as control group, which would be affected similarly if specific hospitals began to upload procedure codes more completely after the DIP reform. Additionally, using uninsured inpatients as control group made our findings exempt from the effects of other concurrent events or hospital management changes that would affect all the inpatients indiscriminately.

 Our findings provide several policy implications. First, the DIP reform, by using differentiated payment in place of fixed rate per admission, incentivized hospitals to pay more attention to severe patients and advanced treatments. Although it may promote medical technology advancement and quality of care, we should also be wary of supplier-induced demand and potential inefficiency. Second, the average DIP weight can be decomposed to identify unusual changes in diagnosis and treatment behavior among providers, which can be used to monitor potential misconduct and facilitate better implementation of the DIP system. Thirdly, hospitals responded to the DIP reform primarily by increasing treatment intensity, especially in specific disease categories with multiple treatment choices. Policy-makers could accordingly determine a set of diseases that require a higher level of supervision. Finally, primary hospitals have relatively weak capability to adapt to the DIP reform, making them disadvantaged in the regional competition. Policy-makers should be aware of the potential financial risks among primary hospitals and explore solutions to induce hierarchical healthcare delivery system rather than pure competition among hospitals of different levels.

 This study had several limitations. First, although the uninsured inpatients were used as control group in the ITS analyses to adjust for events that affect all the inpatients in G city similarly during the study period, it was hard to avoid confounding events that have different impacts on insured inpatients and uninsured inpatients. Second, it could not be determined whether the change in treatment behavior is reasonable. The increased treatment intensity may be a combined effect of releasing of previous unmet demands as well as supplier-induced demand, over-treatment, and vicious competition for DIP weights among hospitals. Third, the long-term impact of DIP reform is still unknown. The average weight could not always increase, even if the observed growth did not slow down during the first two years after the policy. Therefore, longer-term data is required for future analysis. Finally, although there was no significant change in average diagnostic weight, it only indicated that there is no systematic overall upcoding. However, the possibility of upcoding in some specific hospitals and diseases such as low-birth-weight newborns could not be excluded, thus further research is needed.

## Conclusions

 By differentiating payments for cases severity, DIP induced hospitals to shift their focus from volume to weight of inpatients. Instead of diagnostic upcoding, hospitals responded to the DIP reform primarily by increasing treatment intensity. Primary hospitals may face financial risks under regional competition.

## Acknowledgements

 We acknowledge the assistance of Guangzhou Healthcare Security Administration in providing data and materials. We are also thankful to Mengyun Sui, Hongzheng Li, and Long Xue from the School of Public Health, Fudan University for assistance in data cleaning.

## Ethical issues

 This study was approved by the institutional review board of the School of Public Health, Fudan University (IRB #2020-TYSQ-03-20).

## Conflicts of interest

 Authors declare that they have no conflicts of interest.

## Disclaimer

 The opinions expressed in this paper are those of the authors alone and do not necessarily reflect the official policy or position of *International Journal of Health Policy and Management*, its editors, or any affiliated institutions.

## Supplementary files

Supplementary file 1 contains a numerical example for weight calculation, detailed explanation of controlled interrupted time series design, Tables S1-S3, and Figure S1-S5.
