# Supplementary material for: Impact of the Diagnosis-Intervention Packet Payment Reform on Provider Behavior in China: A Controlled Interrupted Time Series Study
Source: Int J Health Policy Manag. 2024 Dec 19;13:8463. doi: 10.34172/ijhpm.8463 (PMC11806229; doi:10.34172/ijhpm.8463)
Supplement: Supplementary file 1 — contains a numerical example for weight calculation, detailed explanation of controlled interrupted time series design, Tables S1-S3, and Figure S1-S5. [file ijhpm-13-8463-s001.pdf]

Article title: Impact of the Diagnosis-Intervention Packet Payment Reform on Provider Behavior in China: A Controlled Interrupted Time Series Study

Journal name: International Journal of Health Policy and Management (IJHPM)

Authors' information: Ruixin Wang[1]¶, Jiaqi Yan[1]¶, Xinyu Zhang[1], Mengcen Qian[1], Xiaohua Ying[1,2]*

[1]School of Public Health, Fudan University, Shanghai, China.

[2]Key Laboratory of Health Technology Assessment (Fudan University), Ministry of Health, Shanghai, China.

***Correspondence to:** Xiaohua Ying; Email: xhying@fudan.edu.cn

¶ Both authors contributed equally to this paper.

Citation: Wang R, Yan J, Zhang X, Qian M, Ying X. Impact of the diagnosis-intervention packet payment reform on provider behavior in China: A controlled interrupted time series study. Int J Health Policy Manag. 2024;13:8463. doi:10.34172/ijhpm.8463

Supplementary file 1

A numerical example for weight calculation

Detailed explanation of controlled interrupted time series design

Table S1: Controlled interrupted time series (ITS) estimates other than β6 and β7

Table S2: Controlled interrupted time series (ITS) estimates of average treatment weight among major disease categories

Table S3: Estimates of average treatment weight among major disease categories stratified by age groups

Figure S1: Monthly changes in inpatient volume, average DIP weight, average diagnostic weight, and average treatment weight in insured inpatients compared to uninsured inpatients among tertiary hospitals in G city, 2016-2019

Figure S2: Monthly changes in inpatient volume, average DIP weight, average diagnostic weight, and average treatment weight in insured inpatients compared to uninsured inpatients among secondary hospitals in G city, 2016-2019

Figure S3: Monthly changes in inpatient volume, average DIP weight, average diagnostic weight, and average treatment weight in insured inpatients compared to uninsured inpatients among primary hospitals in G city, 2016-2019

Figure S4: Monthly changes in average treatment weight among insured and uninsured inpatients with (A) certain conditions originating in the perinatal period and (B) external causes of morbidity and mortality in G city, 2016-2019

Figure S5: Correlation between the number of DIP groups under each disease category and trend effects of average treatment weight based on controlled interrupted time series analyses

A numerical example for weight calculation

Suppose we have the following inpatient cases. For starters, we assign DIP group and weights for all the cases consistently based on the 2018 rule, no matter whether it's after the introduction of the DIP reform (Jan 2018) or not.

Case ID	Discharge month	Primary diagnosis	Procedure	DIP group	DIP weight in 2018
1	Jul 2016	A15.0 Tuberculosis of lung	33.2201 Conservative treatment (Fiberoptic bronchoscopy)	A15.0: 33.2201	943
2	Nov 2019	A15.0 Tuberculosis of lung	33.2201 Conservative treatment (Fiberoptic bronchoscopy)	A15.0: 33.2201	943
3	Sep 2017	A15.0 Tuberculosis of lung	39.7912 Endobronchial arterial embolization	A15.0: 39.7912	1216
4	Mar 2018	A15.0 Tuberculosis of lung	39.7912 Endobronchial arterial embolization	A15.0: 39.7912	1216
5	Jul 2016	I21.0 Acute transmural myocardial infarction of anterior wall	36.0701 Drug-eluting coronary stent implantation	I21.0: 36.0701	3357
6	Feb 2019	I21.0 Acute transmural myocardial infarction of anterior wall	36.0701 Drug-eluting coronary stent implantation	I21.0: 36.0701	3357
7	Apr 2016	I21.0 Acute transmural myocardial infarction of anterior wall	37.6101 Intra-aortic balloon reversal (IABP)	I21.0: 37.6101	7020
8	Mar 2018	I21.0 Acute transmural myocardial infarction of anterior wall	37.6101 Intra-aortic balloon reversal (IABP)	I21.0: 37.6101	7020

<u>Average DIP weight</u>

For each month, we pool all the cases together and calculate the average DIP weight. For instance, assuming that the following data represents all the inpatient cases in Jul 2016 and Mar 2018, the average DIP weight for Jul 2016 is (943*200+3357*1000) / (200+1000) = 2954.67, while the average DIP weight for Mar 2018 is (1216*300+7020*800) / (300+800) = 5437.09.

Discharge month	DIP group	DIP weight in 2018	Monthly cases
Jul 2016	A15.0: 33.2201	943	200
Jul 2016	I21.0: 36.0701	3357	1000
Mar 2018	A15.0: 39.7912	1216	300
Mar 2018	I21.0: 37.6101	7020	800

Average diagnostic weight

First of all, we pool the cases before the DIP reform (2016-2017) together as a cross-sectional data, categorize them by their primary diagnosis, and calculate the average DIP weight for each diagnosis ("diagnostic weight"). The diagnostic weight captures the pre-DIP composition of various treatments within each diagnosis, reflecting its innate property of resource consumption. For instance, "A15.0 Tuberculosis of lung" (diagnostic weight: 1011.25) is believed to consume less resource than "I21.0 Acute transmural myocardial infarction of anterior wall" (diagnostic weight: 4765.85).

Primary diagnosis	DIP group	DIP weight in 2018	Total cases during 2016-2017	Diagnostic weight
A15.0 Tuberculosis of lung	A15.0: 33.2201	943	3000	(943*3000+1216*1000)/(3000+1000)= 1011.25
	A15.0: 39.7912	1216	1000	
I21.0 Acute transmural myocardial infarction of anterior wall	I21.0: 36.0701	3357	8000	(3357*8000+7020*5000)/(8000+5000) =4765.85
	I21.0: 37.6101	7020	5000	

Note: the numbers are hypothetical, and there are many more DIP groups under each diagnosis.

Then we move on to calculate the "average diagnostic weight" (city-monthly level, time-varying), different from "diagnostic weight" (diagnosis-specific, time-invariant). We calculate the counts of inpatient cases for each diagnosis in each month, multiple it by its "diagnostic weight", sum over all the diagnoses, then divided by the total inpatient volume in G city in that month.

Discharge month	Primary diagnosis	Diagnostic weight	Monthly cases	Average diagnostic weight
Jul 2016	A15.0	1011.25	1000	(1011.25*1000+4765.85*2000) / (1000+2000) = 3514.32
	I21.0	4765.85	2000	
Mar 2018	A15.0	1011.25	1500	(1011.25*1500+4765.85*3500) / (1500+3500) = 3639.47
	I21.0	4765.85	3500	

In this example, the share of I21.0 (higher diagnostic weight compared to A15.0) is higher in Mar 2018 than it was in Jul 2016 (70% vs. 66.7%), rendering Mar 2018 with higher average diagnostic weight.

Average treatment weight

First, we calculate the average DIP weight within each diagnosis for each month, which is "treatment weight" (diagnosis-month level).

Discharge month	Primary diagnosis	DIP group	DIP weight in 2018	Monthly cases	Treatment weight
Jul 2016	A15.0 Tuberculosis of lung	A15.0: 33.2201	943	200	(943*200+1216*250) / (200+250) = 1094.67
		A15.0: 39.7912	1216	250	
	I21.0 Acute transmural myocardial infarction of anterior wall	I21.0: 36.0701	3357	1000	(3357*1000+7020*700) / (1000+700) = 4865.29
		I21.0: 37.6101	7020	700	
Mar 2018	A15.0 Tuberculosis of lung	A15.0: 33.2201	943	250	(943*250+1216*300) / (250+300) = 1091.91
		A15.0: 39.7912	1216	300	
	I21.0 Acute transmural myocardial infarction of anterior wall	I21.0: 36.0701	3357	1100	(3357*1100+7020*800) / (1100+800) = 4899.32
		I21.0: 37.6101	7020	800	

Next, we calculate the total count of inpatient cases during 2016-2017 (pre-DIP) for each diagnosis, considering it as the natural composition of different disease diagnoses. We compute the weighted average of pre-DIP cases and "treatment weight" for each diagnosis, to obtain the "average treatment weight" in G city for each month.

Discharge month	Primary diagnosis	Total cases during 2016-2017	Treatment weight for the current month	Average treatment weight
Jul 2016	A15.0 Tuberculosis of lung	4000	1094.67	(1094.67*4000+4865.29*13000)/(4000+13000)=3978.09
	I21.0 Acute transmural myocardial infarction of anterior wall	13000	4865.29	
Mar 2018	A15.0 Tuberculosis of lung	4000	1091.91	(1091.91*4000+4899.32*13000)/(4000+13000)=4003.46
	I21.0 Acute transmural myocardial infarction of anterior wall	13000	4899.32	

In this hypothetical example, the increase of average treatment weight (3978.09 → 4003.46) is majorly caused by the increasing share of the expensive "37.6101 Intra-aortic balloon reversal (IABP)" treatment among inpatients with "I21.0 Acute transmural myocardial infarction of anterior wall" (41.2% → 42.1%), which outstrips the decrease caused by cheaper treatment of "A15.0 Tuberculosis of lung" (the share of the expensive "39.7912 Endobronchial arterial

embolization" decreases from 55.6% to 54.5%), due to the larger share of inpatients with "I21.0 Acute transmural myocardial infarction of anterior wall" compared to "A15.0 Tuberculosis of lung" (76.47% vs 23.53%).

As highlighted in red, the difference between "average diagnostic weight" and "average treatment weight" lies in that, the former is composed of time-invariant weight for each diagnosis and time-varying case proportion across diagnoses, while the latter is composed of time-invariant case proportion across diagnoses and time-varying weight for each diagnosis (due to the time-varying composition of various treatments within each diagnosis). The changes in average diagnostic weight and average treatment weight are independent of each other, and either of them can lead to the changes in average DIP weight.

Detailed explanation of controlled interrupted time series design

The controlled interrupted time series (ITS) model was specified as:

$$Y_t = \beta_0 + \beta_1 T_t + \beta_2 DIP_t + \beta_3 DIP_t T_t + \beta_4 Insured + \beta_5 Insured T_t + \beta_6 Insured DIP_t + \beta_7 Insured DIP_t T_t + \alpha X_t + \epsilon_t$$

where Y_t represents the aggregated outcome variables in G city in month t, including inpatient volume, average DIP weight, average diagnostic weight, and average treatment weight. T_t is a continuous month count from January 2016 to December 2019. DIP_t is a dummy variable, which equals 0 before the DIP reform (January 2016 to December 2017) and equals 1 after the reform (January 2018 to December 2019). $Insured$ is a dummy variable, which equals 0 for uninsured inpatients and equals 1 for insured inpatients. X_t denotes a series of aggregated covariates, including average age, proportion of male, average Charlson Comorbidity Index (CCI), and seasonality (three dummies for the second to fourth quarters). ϵ_t is the error term.

	pre-DIP<br>(DIP_t=0)	post-DIP<br>(DIP_t=1)
Uninsured<br>($Insured$=0)	$\beta_0 + \beta_1 T_t + \alpha X_t + \epsilon_t$	$\beta_0 + \beta_1 T_t + \beta_2 + \beta_3 T_t + \alpha X_t + \epsilon_t$
Insured<br>($Insured$=1)	$\beta_0 + \beta_1 T_t + \beta_4 + \beta_5 T_t + \alpha X_t + \epsilon_t$	$\beta_0 + \beta_1 T_t + \beta_2 + \beta_3 T_t + \beta_4 + \beta_5 T_t + \beta_6 + \beta_7 T_t + \alpha X_t + \epsilon_t$

As shown in the table above, the intercept β_0 represents the baseline level of the outcome variable, and β_1 is the monthly trend of the outcome variable among uninsured inpatients before the DIP reform. β_2 and β_3 are changes in the level and slope of the outcome variable among uninsured inpatients after the reform, respectively. β_4 indicates the difference in baseline level of the outcome variable between the insured and uninsured inpatients, and β_5 indicates the slope difference between the insured and uninsured inpatients in the pre-DIP period. β_6 denotes the difference in immediate level changes between the insured and uninsured inpatients after the DIP reform, and β_7 denotes the difference in monthly trend changes between the insured and uninsured inpatients after the DIP reform.

Intuitively, the controlled interrupted time series design can be construed as a 4-piece segmented regression (uninsured*pre, insured*pre, uninsured*post, insured*post), the main body for each piece is $Y_t = \beta_{\square} + \beta_{\square} T_t$, and the two binary variables $Insured$ and DIP_t were used to indicate the differences across the 4 pieces. By subtracting the pre-trend of the insured and pre-post changes in the uninsured from the insured*post piece, we can obtain the immediate step change (β_6) and trend change (β_7) for the insured inpatients given the DIP compared to what they would have experienced if there weren't the DIP reform.

Table S1. Controlled interrupted time series (ITS) estimates other than β6 and β7

	**β0**	**β1**	**β2**	**β3**	**β4**	**β5**
**Overall**						
Inpatient Volume	38520.935***	467.286*	-108.009	-1273.861***	36642.845***	1516.622***
Average Weight	379.771*	-0.855	16.203	3.655***	-20.773	2.019**
Average Diagnostic Weight	470.428***	-0.854	10.539	1.649**	-5.311	1.016
Average Treatment Weight	734.830***	0.045	-1.304	1.556***	92.906***	0.735
**Tertiary hospitals**						
Inpatient Volume	32594.906***	269.725	-2031.396	-694.007***	32465.902***	1332.459***
Average Weight	685.233***	-1.369**	34.579**	2.251***	33.301	2.913***
Average Diagnostic Weight	621.961***	-1.299**	24.254**	0.660	13.561	1.467**
Average Treatment Weight	921.192***	0.034	1.507	1.458***	82.174***	1.019*
**Secondary hospitals**						
Inpatient Volume	5864.193***	122.840**	2180.771*	-491.975***	3798.262***	54.502
Average Weight	202.315***	1.806*	-12.820	2.203*	-108.320***	-0.807
Average Diagnostic Weight	210.695***	1.494**	-10.657	1.465*	-66.453**	-0.868
Average Treatment Weight	535.799***	0.793*	4.033	-2.583***	140.064***	-0.721
**Primary hospitals**						
Inpatient Volume	290.080*	71.433***	-268.674	-82.495***	342.399	132.652***
Average Weight	463.360***	-0.527	54.453*	1.330	-8.265	1.712
Average Diagnostic Weight	272.238***	1.987***	34.034**	-3.470***	-23.875	0.576
Average Treatment Weight	423.253***	0.757	38.502**	-0.979	115.091*	1.457

Note: * $p<0.05$; ** $p<0.01$; *** $p<0.001$

Table S2. Controlled interrupted time series (ITS) estimates of average treatment weight among major disease categories

Major disease categories	β1	β2	β3	β4	β5	β6	β7
Certain infectious and parasitic diseases (A00–B99)	0.142	14.806	-0.617	-15.305	0.281	-9.187	1.870*
Neoplasms (C00–D48)	-1.581	-43.675	7.233***	-52.112	0.537	69.149	1.122
Diseases of the blood and blood-forming organs and certain disorders involving the immune mechanism (D50–D89)	-0.074	27.599	2.219	9.401	1.115	-23.639	0.829
Endocrine, nutritional and metabolic diseases (E00–E90)	0.574*	18.504***	-1.319***	32.922***	-0.688	-0.476	2.080***
Mental and behavioural disorders (F00–F99)	0.059	1.502	-0.468**	14.222***	0.208	-14.610***	0.530*
Diseases of the nervous system (G00–G99)	1.336**	30.445*	-0.451	0.969	-0.718	-14.891	2.665***
Diseases of the eye and adnexa (H00–H59)	2.625***	7.683	-5.197***	53.037***	-2.714***	-7.116	5.216***
Diseases of the ear and mastoid process (H60–H95)	0.228	45.131	2.675	-55.346	1.261	-110.288*	-2.953
Diseases of the circulatory system (I00–I99)	-1.713	72.081*	6.022**	2.814	2.822*	-46.328	1.423
Diseases of the respiratory system (J00–J99)	0.350***	-11.271***	-0.282	169.207***	-0.183	16.089***	0.804**
Diseases of the digestive system (K00–K93)	-0.69	1.567	2.006**	-66.688***	0.752	2.812	1.015
Diseases of the skin and subcutaneous tissue (L00–L99)	-1.750***	0.462	1.887***	15.502	1.763***	-16.193*	-1.141*
Diseases of the musculoskeletal system and connective tissue (M00–M99)	-1.072	32.854*	2.861*	-149.617**	3.406*	-35.468	2.521
Diseases of the genitourinary system (N00–N99)	-0.680	9.688	2.338***	50.313*	0.619	4.302	0.458
Pregnancy, childbirth and the puerperium (O00–O99)	-0.328***	-5.919**	1.152***	41.877***	-0.099	1.697	-0.173
Congenital malformations, deformations and chromosomal abnormalities (Q00–Q99)	1.031	-46.826	-2.62	136.695***	-0.874	42.21	2.365
Symptoms, signs and abnormal clinical and laboratory findings, not elsewhere classified (R00–R99)	-0.787***	1.738	0.002	74.633***	0.411	14.557	0.35
Injury, poisoning and certain other consequences of external causes (S00–T98)	-0.505	38.687**	2.367*	-3.601	-1.526	4.385	6.263**
Factors influencing health status and contact with health services (Z00–Z99)	-0.055	-5.89	0.969*	32.744***	0.895*	-1.296	2.254***

Note: * $p<0.05$; ** $p<0.01$; *** $p<0.001$

Table S3. Estimates of average treatment weight among major disease categories stratified by age groups

Major disease category	Immediate step change in average treatment weight					Trend change in average treatment weight				
	**age <30**	**age 30~45**	**age 45~60**	**age 60~75**	**age ≥75**	**age <30**	**age 30~45**	**age 45~60**	**age 60~75**	**age ≥75**
Certain infectious and parasitic diseases (A00–B99)	-2.727	-0.288	-0.577	-3.959	-1.595	1.725	1.245	0.884	1.377	1.216
Neoplasms (C00–D48)	13.925	4.052	5.949	1.299	1.762	3.615	4.366	4.662	5.069	4.364
Diseases of the blood and blood-forming organs and certain disorders involving the immune mechanism (D50–D89)	-19.444	-27.779	-20.338	-21.937	-27.353	0.846	1.227	0.886	0.983	0.914
Endocrine, nutritional and metabolic diseases (E00–E90)	-0.959	-1.892	-4.074	-1.448	-4.050	1.966***	1.873***	1.941***	1.972***	1.962***
Mental and behavioural disorders (F00–F99)	-13.678***	-13.241***	-13.640***	-13.690***	-13.095***	0.216	0.257	0.268	0.338	0.415
Diseases of the nervous system (G00–G99)	-18.603*	-15.969	-13.845	-14.736	-14.555	2.696***	2.582***	2.639***	2.516***	2.295***
Diseases of the eye and adnexa (H00–H59)	-3.759	-1.206	-3.977	-3.450	-3.518	6.167***	5.909***	5.325***	6.073***	6.242***
Diseases of the ear and mastoid process (H60–H95)	-94.351	-104.504	-111.229	-112.725	-106.326	-2.409	-2.898	-2.679	-4.017	-3.138
Diseases of the circulatory system (I00–I99)	-34.715	-59.225	-45.231	-37.869	-42.369	1.425	1.086	2.170	2.728	2.198
Diseases of the respiratory system (J00–J99)	17.058***	15.886***	16.573***	14.043***	16.056***	0.831**	0.794**	0.730**	0.799**	0.767**
Diseases of the digestive system (K00–K93)	-1.852	0.308	7.739	3.586	-1.741	0.925	1.103	1.099	1.137	0.994
Diseases of the skin and subcutaneous tissue (L00–L99)	-17.039*	-18.033*	-15.674*	-14.066	-12.639	-1.163	-0.869	-1.076	-1.252*	-1.186
Diseases of the musculoskeletal system and connective tissue (M00–M99)	-51.723**	-65.029**	-36.367	-50.047**	-50.533**	4.552**	4.202**	4.193**	4.154**	3.715*
Diseases of the genitourinary system (N00–N99)	6.323	4.078	9.695	7.578	5.300	0.177	0.312	0.480	0.292	0.413
Pregnancy, childbirth and the puerperium (O00–O99)	0.671	0.461	0.408	–	–	-0.436	-0.472	-0.428	–	–
Congenital malformations, deformations and chromosomal abnormalities (Q00–Q99)	42.842	36.762	35.271	21.908	–	2.644	3.334	3.169	2.890	–
Symptoms, signs and abnormal clinical and laboratory findings, not elsewhere classified (R00–R99)	14.942	14.203	17.479*	14.818	16.272	0.476	0.169	0.460	0.370	0.305
Injury, poisoning and certain other consequences of external causes (S00–T98)	11.111	7.561	13.023	6.470	11.441	5.515**	5.852**	4.504*	5.504**	5.486**
Factors influencing health status and contact with health services (Z00–Z99)	-1.286	-2.562	-1.650	-2.619	-2.008	1.954***	1.692**	2.061***	2.317***	2.135**

Note: * p<0.05; ** p<0.01; *** p<0.001

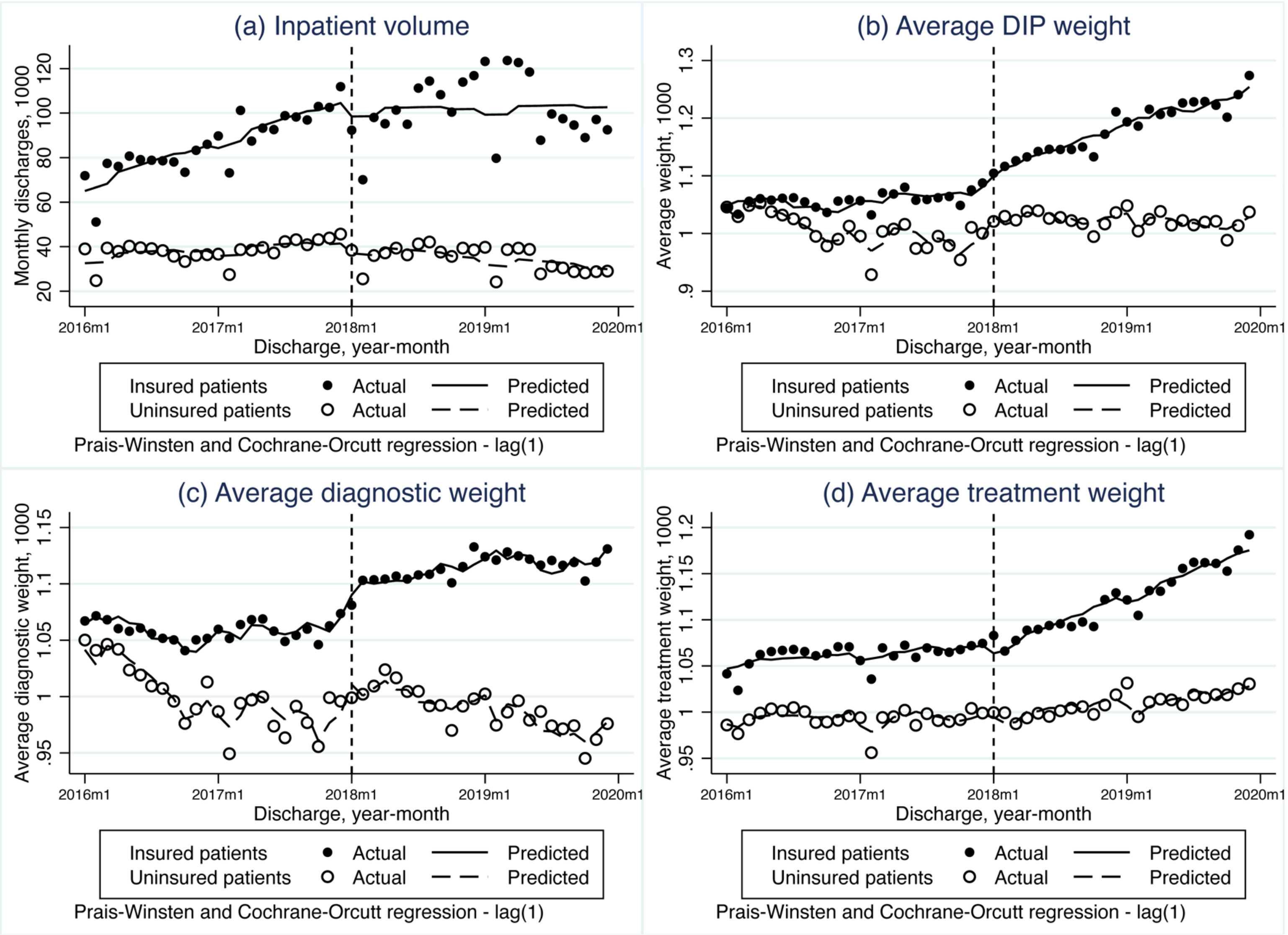

Figure S1. Monthly changes in inpatient volume, average DIP weight, average diagnostic weight, and average treatment weight in insured inpatients compared to uninsured inpatients among tertiary hospitals in G city, 2016-2019

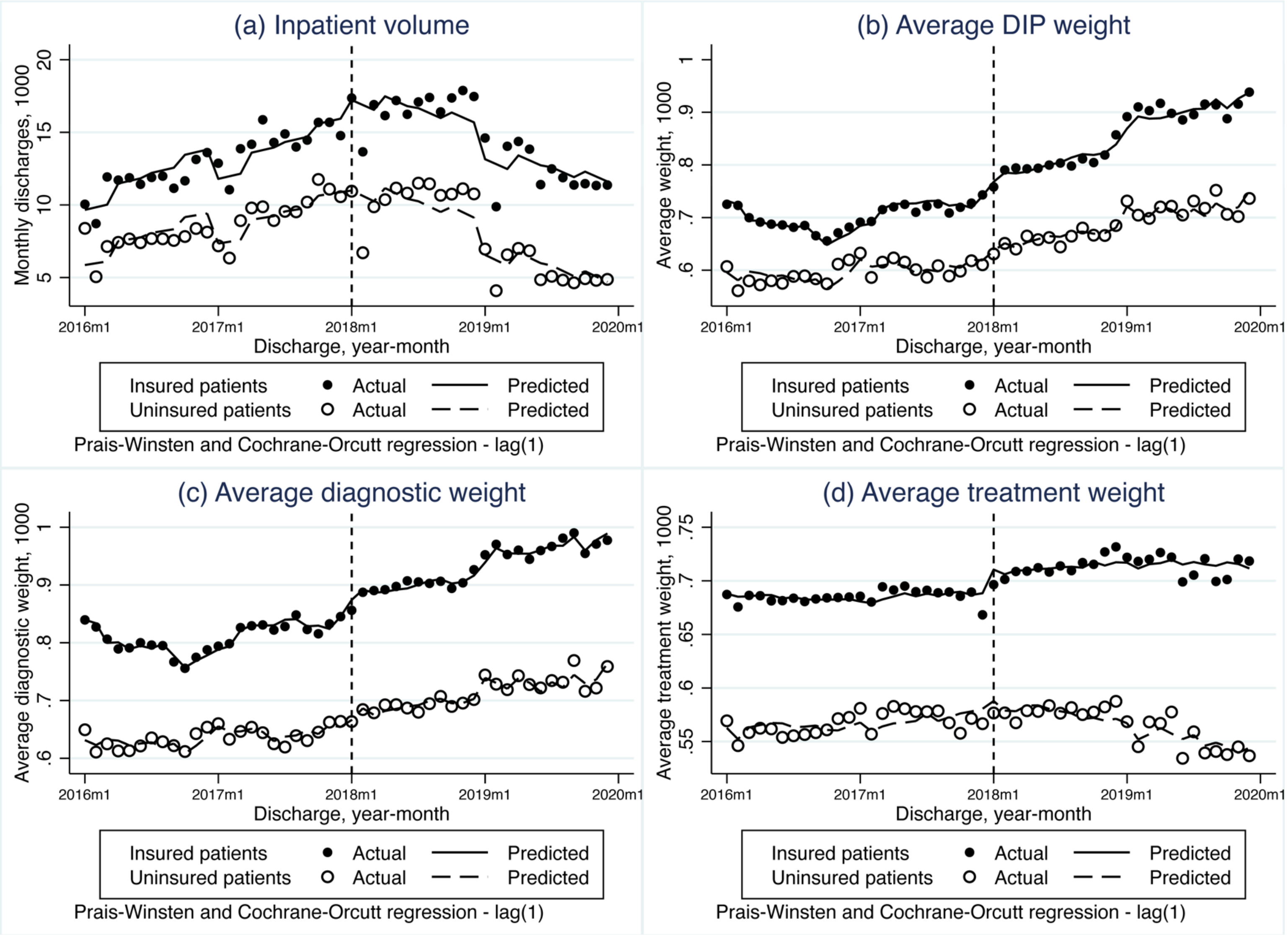

Figure S2. Monthly changes in inpatient volume, average DIP weight, average diagnostic weight, and average treatment weight in insured inpatients compared to uninsured inpatients among secondary hospitals in G city, 2016-2019

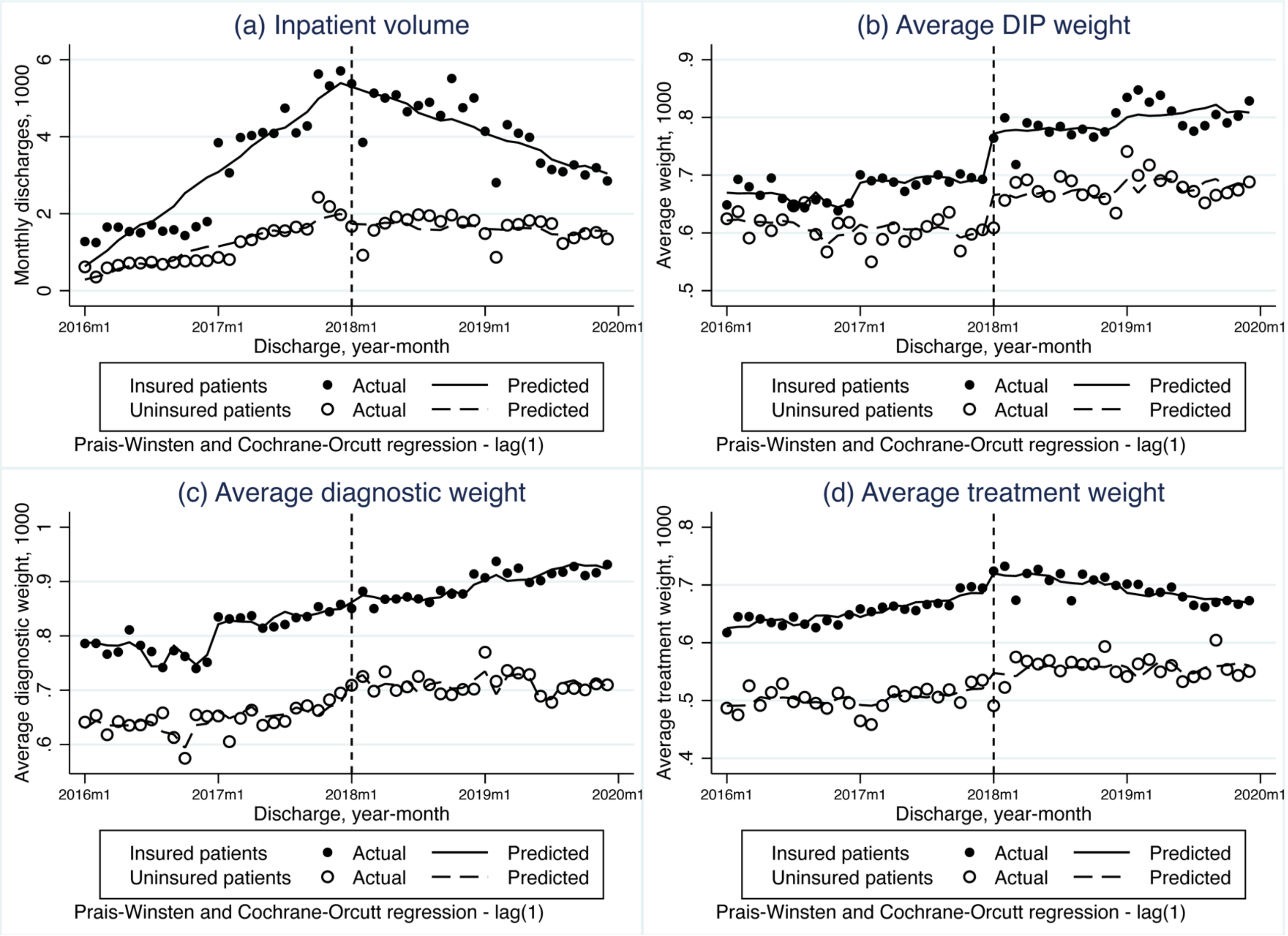

Figure S3. Monthly changes in inpatient volume, average DIP weight, average diagnostic weight, and average treatment weight in insured inpatients compared to uninsured inpatients among primary hospitals in G city, 2016-2019

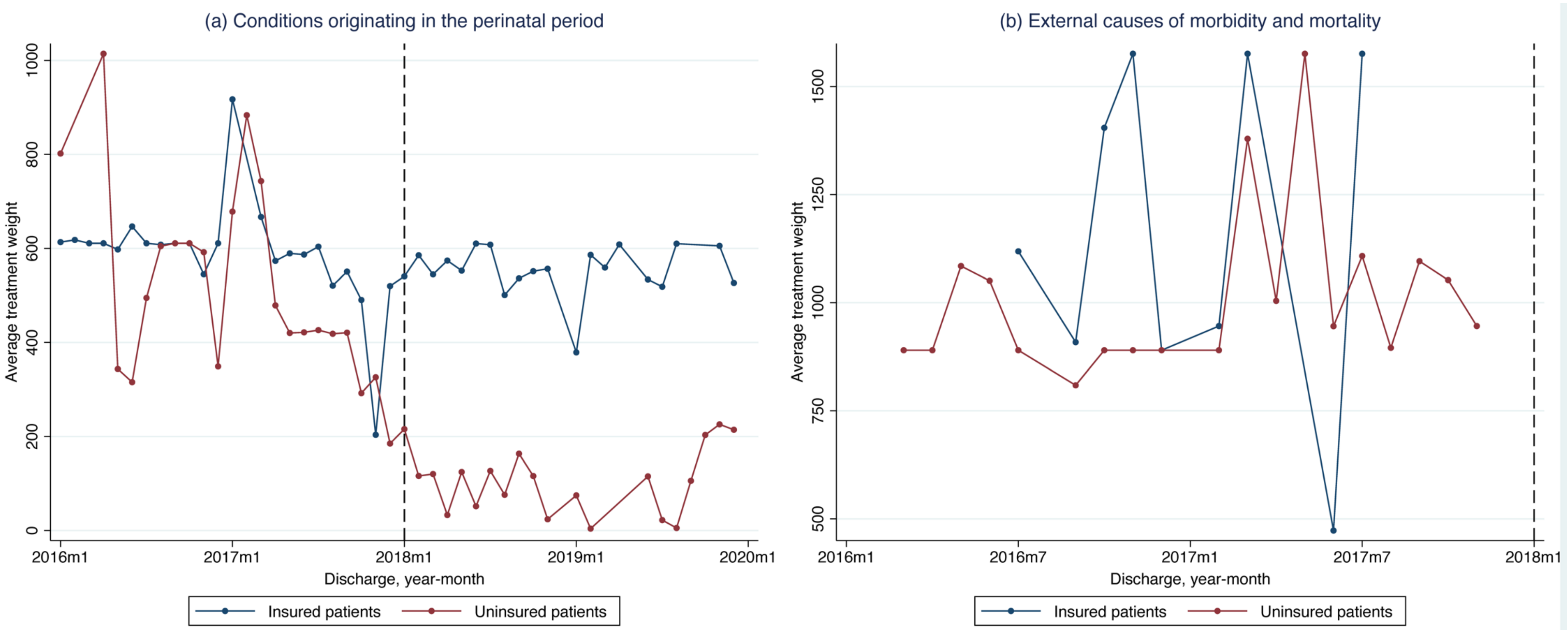

Figure S4. Monthly changes in average treatment weight among insured and uninsured inpatients with (a) certain conditions originating in the perinatal period and (b) external causes of morbidity and mortality in G city, 2016-2019

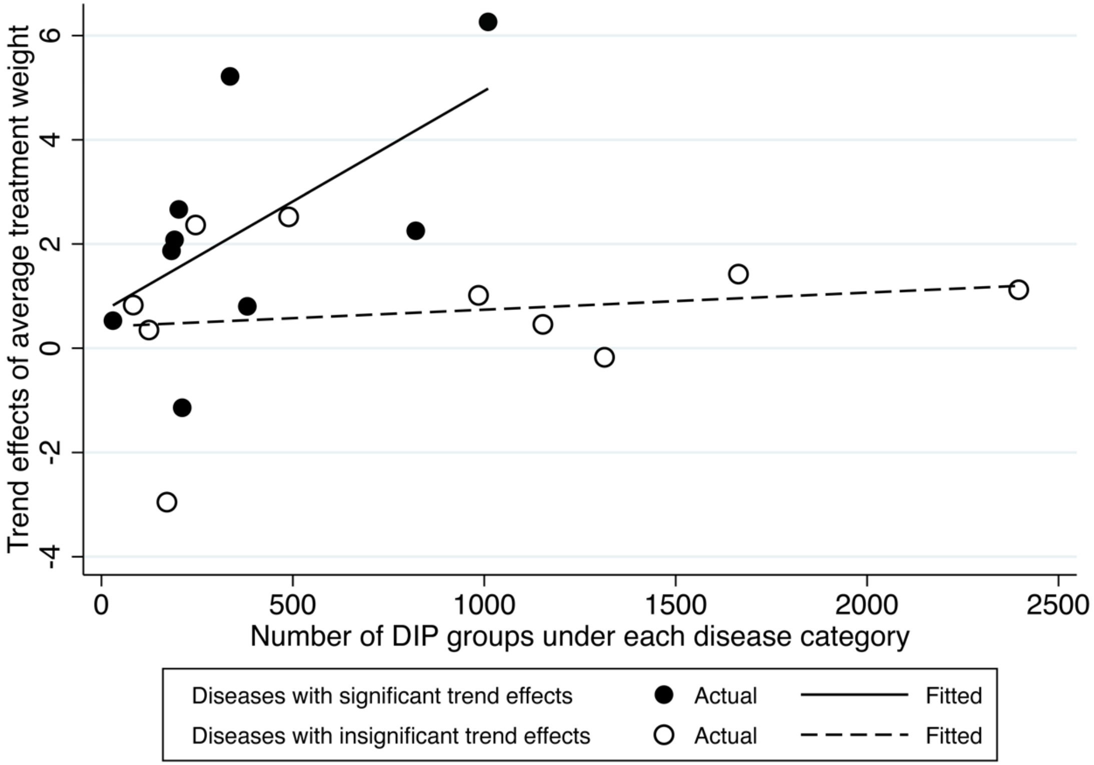

Figure S5. Correlation between the number of DIP groups under each disease category and trend effects of average treatment weight based on controlled interrupted time series analyses
